# Generation of Highly Antioxidant Submicron Particles from *Myrtus communis* Leaf Extract by Supercritical Antisolvent Extraction Process

**DOI:** 10.3390/antiox12020530

**Published:** 2023-02-20

**Authors:** Diego Valor, Antonio Montes, María Calderón-Domínguez, Inass Aghziel, Ismael Sánchez-Gomar, Martín Alcalá, Ma Carmen Durán-Ruiz, Clara Pereyra

**Affiliations:** 1 Department of Chemical Engineering and Food Technology, Faculty of Sciences, University of Cadiz, International Excellence Agrifood Campus (CeiA3), Campus Universitario Río San Pedro, 11510 Cadiz, Spain; 2 Biomedicine, Biotechnology and Public Health Department, Cádiz University, 11002 Cadiz, Spain; 3 Biomedical Research and Innovation Institute of Cadiz (INiBICA), 11009 Cadiz, Spain; 4 Department of Chemistry and Biochemistry, Faculty of Pharmacy, University San Pablo-CEU, CEU Universities, Urbanización Montepríncipe, 28660 Madrid, Spain

**Keywords:** microparticles, supercritical antisolvent, ROS, *Myrtus communis*, carbon dioxide

## Abstract

Submicron particles have been produced from an ethanolic extract of *Myrtus communnis* leaves using supercritical carbon dioxide technology, hereinafter referred to as Supercritical Antisolvent Extraction (SAE). The influence of pressure (9–20 MPa), temperature (308 and 328 K) and injection rate (3 and 8 mL/min) on the particles’ precipitation has been investigated, and it has been confirmed that increases in pressure and temperature led to smaller particle sizes. The obtained particles had a quasi-spherical shape with sizes ranging from 0.42 to 1.32 μm. Moreover, the bioactivity of the generated particles was assessed and large contents of phenolic compounds with a high antioxidant activity were measured. The particles were also subjected to in vitro studies against oxidative stress. The myrtle particles demonstrated cytoprotective properties when applied at low concentrations (1 μM) to macrophage cell lines.

## 1. Introduction

In recent decades, the interest in food, cosmetics and pharmaceutical antioxidants of natural origin with the capacity to replace synthetic antioxidants has increased considerably [1]. Plants have long been a source of exogenous antioxidants, and most of these have a quite high antioxidant capacity [2]. There is a wealth of evidence reporting that the supplementation with exogenous antioxidants or the reinforcement of the body’s oxidative self-defense systems is a powerful strategy to prevent the development of a number of potentially lethal diseases, including neurodegenerative and cardiovascular disorders [3].

A multitude of in vitro trials can be found in the literature, where the antioxidant potential of every plant type has been tested. Thus, some plants produce and accumulate a variety of low weight molecules such as vitamins, phenolic acids, etc., as well as certain secondary metabolites of high molecular weight, such as tannins or phenolic compounds with the antioxidant capacity to act as reductive agents that account for the high bioactive capacity of certain plants [4]. These metabolism reducing agents can be synthesized by plants in response to unfavorable environmental conditions [5].

Oxidative stress is one of the health disorders that can be prevented by treatment with this type of bioactive compound. Oxidative stress is defined as a disturbance in the balance between the production of reactive oxygen species (ROS) and antioxidant defenses [6]. It is, therefore, a consequence of either an excessive generation of free radicals or of a reduced activity of the antioxidant defenses [7]. Antioxidant defenses protect biological systems from ROS toxicity. Enzymes are specific antioxidants that catalyze reactions and neutralize free radicals and reactive oxygen species. Some examples of antioxidant enzymes include, among others, superoxide dismutase (SOD), catalase (CAT), glutathione peroxidase, glutathione-S-transferase, and glucose-6-phosphate dehydrogenase [8,9].

*Myrtus communis* L. is a plant from the Myrtaceae family that is widely distributed in the Mediterranean area and in the Middle East that grows wild in mountainous regions [10]. Although this species was valued in ancient times for its ornamental and aromatic potential, it is currently mostly used for its medicinal properties, and is used in the food and pharmaceutical industries. In recent years, the pharmacological properties of its essential oil, such as its anti-hyperglycemic activities, antimicrobial properties, and antioxidant activity, have been explored in depth. The essential oils extracted from its leaves are used in the perfumery and food industries, while its leaves and berries are mainly used as a source of antioxidants and for the production of liqueurs [11]. 

Specifically, crude extracts and phenolic fractions of *Myrtus communis* were found to have inhibitory capacity against enzymes such as 5-lipoxygenase, cyclooxygenase-1 and acetylcholinesterase, which are involved in several human diseases [12]. In addition, a MTT (3-(4,5-dimethylthiazol-2-yl)-2,5-diphenyltetrazolium bromide) metabolism assay demonstrated its efficacy against the proliferation of PC12 cancer cells and its safety with regard to normal cells [13].

The characterization and quantification of different samples of *Myrtus communis* L. highlighted that myrtle leaves are particularly rich in phenolic compounds, especially hydrolysable tannins and flavonoids, which makes the use of its alcoholic extracts interesting for the cosmetic, nutraceutical and pharmaceutical fields [14]. Phenolic compounds are the most abundant secondary metabolites in plants. These are found naturally in fruits, vegetables, cereals, roots and leaves, among other plant products [15]. Phenolic compounds have at least one aromatic ring in their chemical structure to which one or more hydroxyl groups are attached. They can be divided into several classes, including flavonoids, phenolic acids, tannins, stilbenes, lignans, coumarins and quinones [16]. According to the study by d’Urso et al. the main phenolic compounds characterized in myrtle leaves extract were, among others, galloyl-hexahydroxyphenyl-glucose isomers, digaloylquinic acid, myricetin, quercetin, and its derivatives.

Plant extracts are generally studied and marketed in liquid form. This presents a series of drawbacks such as heavy transport costs, a high biodegradation rate, and a frequently poor compound content per mass of liquid depending on the extraction method, in addition to the issues related to the final product as a consequence of the use of organic solvents for compound extraction [17,18,19]. When these challenges are overcome, plant extracts can become much more effective as solid formulations in the form of micro/nanoparticles [20]. The field of micro/nanoparticle technology has an enormous potential for therapeutical purposes, since it can increase the bioavailability to the bioactive products in the body. Some examples are the extracts from *Mimosa pudica* leaves [21] or the raw material that our work is focused on, i.e., the extract from *Myrtus communis* L. [22].

There are a multitude of techniques to obtain fine particles from extracts such as spray-drying or freeze-drying that are well documented in the literature [23,24,25]. More novel techniques, such as those involving the use of supercritical fluids (SCFs), remain to be more deeply investigated. These techniques, depending on the various nucleation and growth mechanisms involved in particle precipitation processes, can be classified into different groups according to the role to be played by the pressurized fluid, i.e., either solvent or anti-solvent [26]. Carbon dioxide (CO_2_) is the compound that is most often used for high pressure precipitation processes under supercritical conditions. CO_2_ is not only considered an environmentally friendly compound, but it is also non-flammable, non-toxic, and leaves no residues on the processed samples. Furthermore, it is worth noting that its critical pressure and, in particular, its relatively low critical temperature (Pc = 73.8 bar, Tc = 31.1 °C) do not represent a degradation hazard for the thermosensitive compounds that are expected to be found in most plant extracts [26]. In addition to this, supercritical CO_2_-based micronization techniques allow the generation of regular particles with minimal dimensions. This is particularly difficult to achieve when other more conventional technologies are used [27]. Some of the techniques based on SCFs are Particle form Gas-Saturated Solutions (PGSS), where the pressurized liquid serves as solute; Rapid Expansion of Supercritical Solutions (RESS), in which the fluid acts as solvent; and various anti-solvent processes, such as Supercritical Anti-Solvent (SAS), Gas Anti-Solvent (GAS) or the Aerosol Solvent Extraction System (ASES) [28]. In certain studies, supercritical antisolvent techniques have been used for the precipitation of particles from natural extracts, as in the following examples: the co-precipitation of lycopene extract using polyvinylpyrrolidone (PVP) [29], the formation of *Curcuma xanthorrhiza* extract microparticles [30], the encapsulation of diterpenes from spent coffee grounds [31], microparticles from *Mangifera indica* leaf extract together with a fluorescent marker [32], or the precipitation of *Glycyrrhiza glabra* [33]. In these cases, the process is known as Supercritical Antisolvent Extraction (SAE) [34]. Compared to other techniques for the production of powdered extracts and produce particles, such as spray drying, spray cooling, lyophilization or dry processes, SAE has advantages such as more homogeneous particle size distributions, the prevention of mechanical or thermal degradation of micronized compounds due to the relatively low temperatures that are often used, and the improved elimination of the used organic solvents due to the anti-solvent power of supercritical CO_2_ [35]. It is therefore an appropriate alternative to these more conventional processes for the production of micro or nanoparticles of bioactive compounds [36].

In the aforementioned context, the aim of the current study was to apply a SAE method to produce low size and highly antioxidant particles from *Myrtus communis* leaf extracts and subsequently to evaluate their effect on cell viability and the expression of the antioxidant enzymes superoxide dismutase (Sod1, Sod2) and catalase (Cat) after inducing oxidative stress to a macrophage cell line (RAW 264.7). Moreover, the authors have evaluated the effect on the extracts’ mean particle size and antioxidant activity that is attributable to each of the most influential variables involved in SAE, such as pressure, temperature and extract injection rate.

## 2. Materials and Methods

### 2.1. Materials

The dehydrated *Myrtus communis* leaves were supplied by Valley of Tea. The commercial 2,2-diphenyl-1-picrylhydrazyl (DPPH), MTT, ethanol (99.5%), tert-butyl hydroperoxide (tBHP), Folin–Ciocâlteu reagent (FCR), dimethyl sulfoxide (DMSO) and enzymatic primers of β-actina, catalasa, *Sod1*, *Sod2* were supplied by Sigma–Aldrich (Madrid, Spain). The phosphate-buffered saline (PBS), Dulbecco’s Modified Eagle’s Medium, and penicillin-streptomycin were supplied by Biowest. The CO_2_ (99.8 % minimum purity) was supplied by Linde (Barcelona, Spain). The RAW 264.7 macrophage cell line was supplied by ATCC. The Direct-zol™ RNA MiniPrep kits were supplied by Zymo Research.

### 2.2. Producing the Myrtle Leaf Extract

The myrtle leaf extract was obtained by the maceration extraction method. The extraction was carried out on a 50 g sample of dried and crushed myrtle leaves. Ethanol (99.5%) was the solvent used for 3 h under agitation at a mild temperature of 45 °C, so that the degradation of the compounds present in the leaves would be avoided. Ethanol was used as the organic solvent due to its high solubility in the supercritical phase and therefore was easy to remove from the final product. The ethanolic suspension thus obtained was filtered using a commercial filter paper and collected and stored in darkness at 4 °C prior to assay. In order to achieve the desired total concentration, a rotary evaporator at 45 °C was employed. The final extract to be used for the subsequent processes had 25 mg/mL concentration. This final concentration used for the extract was based on previous work with the SAE technique for the precipitation of plant leaf extracts [32,37].

### 2.3. Particle Formation Process

**Figure 1:**
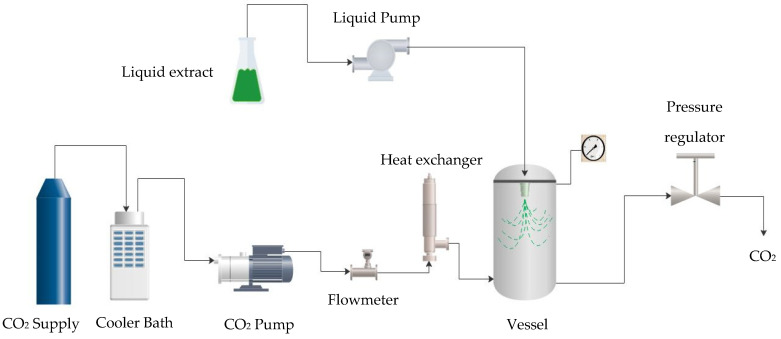
Schematic diagram of precipitation equipment.

The submicron particles from the myrtle leaves extract were obtained by Supercritical Antisolvent Extraction (SAE) by means of a pilot plant built by Thar Technologies (a schematic operational diagram can be seen in Figure 1). This technique consists in putting into contact an organic solution, in this case, an ethanolic extract containing a multitude of organic compounds, with supercritical carbon dioxide. The plant was equipped with a high-pressure pump to inject the CO_2_ into the system, while another pump inserted the ethanolic extract, which had been previously obtained, into a 0.5 L vessel. A cooling bath was used to bring the CO_2_ into a liquid state before it was injected and heated up by the system. The pressure level was controlled by means of an automatic back pressure regulator and the temperature was controlled by a heat exchanger coupled to heating jackets. After the pressure and temperature reached the desired operating levels, the liquid solution was sprayed through a nozzle into the vessel that contains the CO_2_ under supercritical conditions. When the scCO_2_ mixed with the organic solution, it was quickly dissolved, which caused the precipitation of the solutes by antisolvent effect (solution micro-droplets are formed). After the injection of the solution stopped, the supercritical CO_2_ continued to flow for 1 additional hour in order to remove the residual solvent. When this washing step was completed, the CO_2_ flow was interrupted and the vessel was depressurized to atmospheric pressure. The precipitated powder was then ready for collection.

A total of 11 experiments resulted from applying a 2^3^ factorial experimental design with three center points. The effect of pressure and temperature levels as well as the liquid extract injection rate was tested by using two different values for each of these variables. The influence of these parameters on the process was analyzed by means of the software application Statgraphics Centurion XIX. A summary of the experiments is shown in Table 1. The CO_2_ flow rate was maintained at a constant of 25 g CO_2_/min over the precipitation and the additional one-hour washing phases. Extract samples of 30 mL were used for each experiment. 

### 2.4. Scanning Electron Microscopy (SEM)

A FEI Nova NanoSEM 450TM scanning electron microscope (Thermo Fisher, Frankfurt, Germany) was employed to examine the morphology and particle size of the samples using a 10 kV beam voltage. Prior to analysis, the samples were deposited on carbon tape and coated with a 10 nm thick gold layer using the Cressington 208HR Metalliser in order to improve their conductivity.

### 2.5. Analysis of Phenolic Composition

The analysis of the chemical composition of the starting extract and the obtained particles in each performed experiment was performed with an Acquity™ Ultra Performance Liquid Chromatography system (Waters, Milford, MA, USA) coupled to quadrupole-time-of-flight mass spectrometry Xevo™ G2 QTof MS (Waters, Milford, MA, USA). Analyses were processed by the Target Lynx version of MassLynx 4.1 software. A reverse phase column (Acquity UPLC BEH C18, 2.1 × 100 mm, 1.7 μm particle size) from Waters was used for the compound quantification. The column was set at 45 °C, the solvent flow rate was maintained at 0.4 mL/min, and the injection volume was set as 2 μL. The binary system phases were water +0.1% formic acid (solvent A) and methanol (solvent B), with the following phase gradient: initial time, 95% A for 5 min, 30% A from 5 to 6 min, 5% A from 6 to 7 min, and 95% A from 7 to 10 min. 

Electrospray was operated in negative ionization mode and a mass range of 100–1200 Da, using the following ion source parameters: a capillary voltage of 2.5 kV, a cone voltage of 40 V, and a source temperature of 120 °C. Prior to analysis, samples were filtered using a 0.2 μm nylon syringe filter, and the injections were performed in duplicate. Compounds were detected and quantified according to the retention time and using the calibration curves for the different commercial standards, which are shown in Table 2.

### 2.6. Total Polyphenols Content (TPC)

The total phenolic content of the precipitate was analyzed following the Folin-Ciocalteau method described by Singleton and Rossi [38] (with certain modifications) for microplates (Greiner Cellstar^®^ 96 well) [39]. A 12.5 μL aliquot of the main extract and obtained particles from SAE experiments diluted in ethanol to 1000 μg/mL was mixed with 12.5 μL of Folin-Ciocalteu’s reagent and 200 μL distilled water and shaken for 5 min. Subsequently, 25 μL of sodium carbonate solution (20% *w*/*v*) was added and the mixture and stirred for another 5 min. After 60 min in the absence of light at room temperature, absorbance was measured at 725 nm by means of a Synergy HTX multi-mode reader (Aligent, Biotek, VT, USA). The absorbance of the same reaction with ethanol instead of the extract, particles or standard was subtracted from the absorbance of the reaction with the samples. A calibration curve was carried out using gallic acid (20–300 μg/mL, R^2^ = 0.997) and the results were presented as milligrams of gallic acid equivalent per gram of dry sample (mg GAE/g dry sample). The analyses were carried out in duplicate.

### 2.7. Antioxidant Activity

The reductive capacity of the precipitated samples was evaluated by DPPH. The method used was based on the one proposed by Brand-Williams and Scherer [40,41], with modifications for use in microplates. The samples were prepared at concentrations ranging from 31.25 μg/mL to 2000 μg/mL. Aliquots of 7 μL of the particles at different concentrations were taken and mixed with 293 μL of the working solution of DPPH (6 × 10^−5^ mol DPPH/L ethanol). After 2 h in the absence of light, when the reaction was completed, absorbance was measured at 517 nm. Each test was carried out in duplicate. A calibration curve of DPPH (Abs = 11.079·C—0.0278) was performed for the quantification of the antioxidant activity of the precipitates at the end of the process, also measured at 517 nm absorbance. Trolox was used as a positive control for comparison, ethanol as a negative control, and particles without DDPH as a blank.

The percentage of remaining DPPH could be calculated according to the following equation:

(1) $$\%\;\mathrm{DPPH}\;\mathrm{Remaining}=\frac{C_{\mathrm{DPPHt}}}{C_{\mathrm{DPPH}0}}\times 100$$

where C_DPPHt_ is the absorbance at time t and C_DPPH0_ is the initial absorbance. Oxidation inhibition is defined as the percentage of DPPH that remains unreacted:

(2) $$\%\;\mathrm{Oxidation}\;\mathrm{inhibition}=\frac{C_{\mathrm{DPPH}0}-{\;C}_{\mathrm{DPPHt}}}{C_{\mathrm{DPPH}0}}\times 100$$

The IC_50_ value was graphically calculated using a polynomial fitting curve by plotting the sample concentration vs. the remaining % DPPH. Finally, the antioxidant activity index (AAI) was calculated as a function of the IC_50_ value and the final DPPH concentration in the reaction medium according to the following equation:

(3) $$\mathrm{AAI}=\frac{C_{\mathrm{DPPH}}}{{\mathrm{IC}}_{50}}$$

### 2.8. In Vitro Application

#### 2.8.1. Cell Culture and Treatments

Murine RAW 264.7 macrophages were supplied by ATCC and were grown in DMEM supplemented with 10% heat-inactivated FBS and a 1% penicillin-streptomycin mixture at 37 °C with 5% CO_2_ in a humidified atmosphere. In order to induce oxidative stress and cellular damage, cells were treated with 250 μM tert-butyl hydroperoxide (tBHP) dissolved in DMEM directly into the media as a positive control. After one hour, the cells were co-treated with 1, 10, 25, and 50 μM of myrtle leaf extract or particles for 16 h.

#### 2.8.2. Determination of Cell Viability

RAW 264.7 macrophages were seeded in 96-well plates (1.5 × 10^4^ cells/well), cultured for 24 h and treated as described above. The cell viability was then determined with the 3-(4,5-dimethylthiazol-2-yl)-3-(4,5-Dimethyl-2-thiazolyl)-2,5-diphenyl-2H-tetrazolium bromide (MTT) assay (ACROS ORGANICS), as described [42] in triplicate, with each being repeated at least three times. 

#### 2.8.3. Analysis of mRNA Expression by Quantitative Real-Time PCR (qPCR)

The total RNA was isolated from the cultured and treated cells grown in 6-well plates using Direct-zol™ RNA Miniprep (ZYMO Research), and the cDNA was acquired using a PrimeScript™ RT Master Mix (TAKARA) following the manufacturer’s instructions. mRNA relative quantification was performed using a CFX Connect Real-Time PCR Detection System (BioRad) in 10 μL of reaction medium by using 6.5 ng of cDNA, forward and reverse primers at 100 nM each, and a SYBR Green PCR Master Mix Reagent kit (Life Technologies) The primer pairs are shown in Table 3. Each sample was amplified in triplicate for the mRNA of interest, and each assay was repeated at least four times. Relative mRNA level quantification was performed using the ΔΔCt method, and were normalized to those of β-actin and expressed as fold change.

#### 2.8.4. Determining the Antioxidant Capacity and Oxidative Damage

The cell lysates were prepared in a buffer containing 50 mM Tris, pH = 7.4 and 5 mM EDTA. An amount of 5 mM BHT was added to prevent the oxidation of the samples in those aliquots intended to be used to determine the products resulting from the oxidative damage to the proteins.

The Catalase (CAT) activity was measured by monitoring the disappearance of 100 μL of 30 mM hydrogen peroxide at 240 nm over time in a final volume of 1 mL with 50 mM phosphate buffer pH = 7, at 37 °C [43].

The Superoxide Dismutase (SOD) activity assay was based on the inhibition of cytochrome C oxidation in the presence of the superoxide generated by xanthine and xanthine oxidase [44]. Briefly, 50 μL of the diluted sample was combined in a spectrophotometric cuvette with 1 mL of 50 mM phosphate buffer, pH = 7.8 at 37 °C, 50 μL of 0.1 mM cytochrome C, and 250 μL of 0.5 mM xanthine. The reaction was triggered by the adding of 50 μL of 0.02 U/mg protein of xanthine oxidase. The appearance of the oxidized cytochrome C was continuously measured at 546 nm for 2 min.

The Glutathione Peroxidase (Gpx) activity was measured by adapting the method described by Gunzler et al. [45]. A total of 50 μL of the diluted sample catalyzes the oxidation of 20 μL of 0.1 M reduced glutathione (GSH) in the presence of 20 μL of 30 mM tBHP. The resulting oxidized glutathione was then reduced using 20 μL of 50 U/mL glutathione reductase, which uses NADPH + H+ as a cofactor. The disappearance of this reducing power was continuously monitored at 37 °C for 1 min at 340 nm.

Advanced Oxidation Protein Products (AOPP) are uremic toxins generated as a result of the oxidation of proteins by chlorinated oxidants. The AOPPs in the samples were calorimetrically detected by using potassium iodine in an acid environment, as previously described [46]. The AOPP concentrations were expressed as micromoles of Chloramine-T equivalents per mg of analyzed tissue.

The amount of GSH was fluorometrically detected in the presence of O-Phtaldialdehyde (OPD). First, 50 μL of sample were deproteinized using 150 μL of 5% TCA in order to prevent non- specific crossed reactions between the OPD and the cysteine residues. The supernatant was then neutralized with 0.1 M NaOH and incubated with 10 μL of 1 mg/mL OPD. The fluorescent products were measured at Ex = 350 nm/Em = 420 nm, and the results were interpolated from a GSH calibration curve. 

#### 2.8.5. Statistical Analysis

The results are presented as means ± SEM. For each variable, outliers were identified using the Rout test with a Q = 1%. The normality of the distributions was then assessed using the Kolmogorov-Smirnoff test. For Gaussian distributions, a Student’s t-test or one-way Analysis of Variance (ANOVA) followed by a post hoc Tukey’s multiple comparison test was applied. Otherwise, a Mann-Whitney test and Dunn’s multiple comparison test were respectively used. The statistical analyses were performed using the software application Graph-Pad Prism (version 9 for Windows, GraphPad, San Diego, CA, USA) and the statistical differences were considered as significant when *p* < 0.05.

## 3. Results and Discussion

### 3.1. Supercritical Antisolvent Extraction 

#### 3.1.1. Morphology and Particle Size

**Figure 2:**
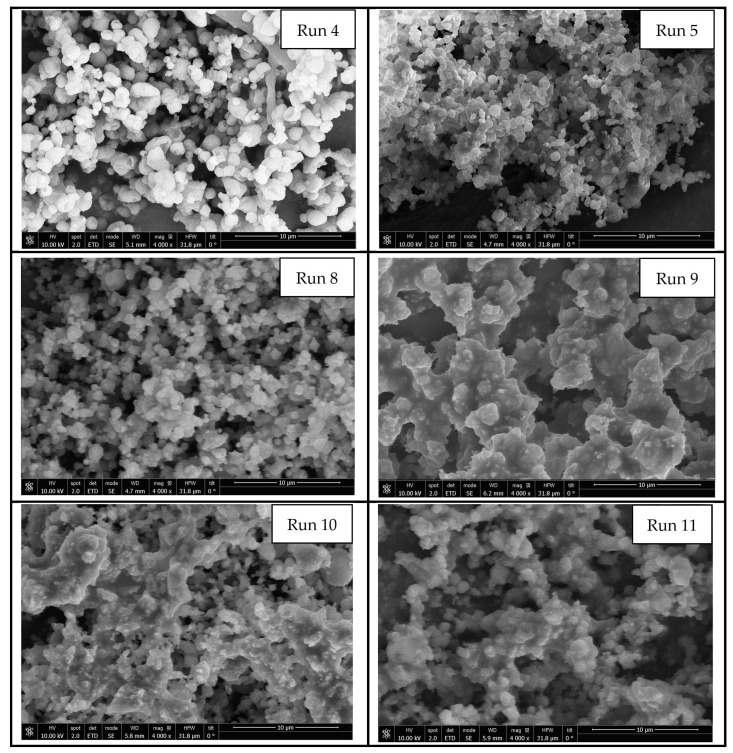
SEM images of the particles precipitated from myrtle leaf extracts.

Most of the performed SAE experiments produced a powder precipitation on the entire inner surface of the vessel. The precipitation of the myrtle leaf extract was carried out by varying the operating conditions in order to determine the appropriate ones to be used for the SAE experiments (Table 1). Pressure and temperature are two of the main parameters influencing morphology and particle size, since they play an important role with respect to the physical state of the solvent-CO_2_ mixture. The injection rate of the extract into the vessel was also investigated because of its likely influence on the mass transfer that occurs during the precipitation stage. Figure 2 shows the scanning electron microscopy images of the precipitated powders. In the successful experiments, 0.42 to 1.32 μm sized quasi-spherical particles connected to each other were obtained. A summary of the results regarding particle size can be seen in Table 3.

**Figure 3:**
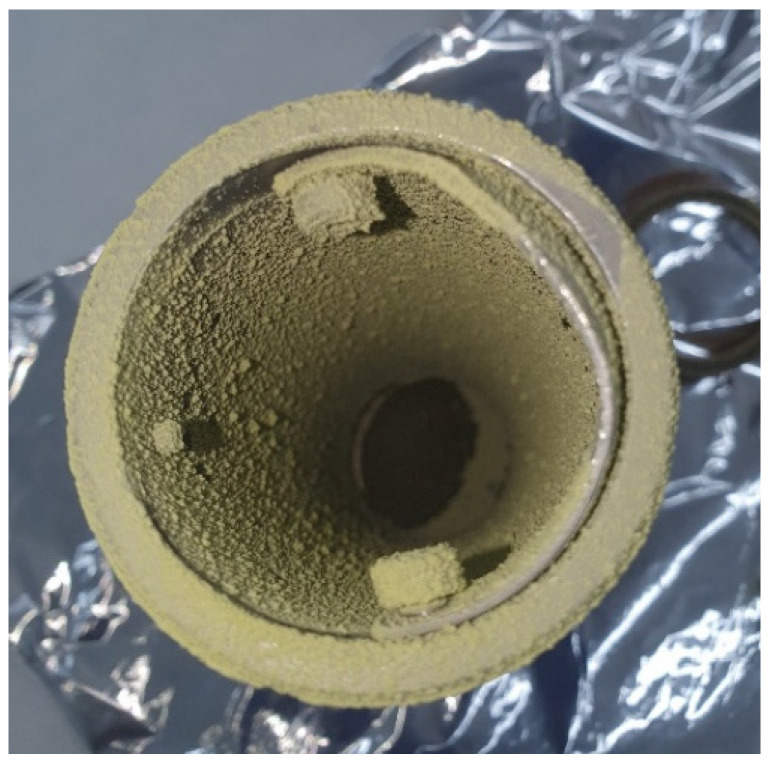
Precipitated powder obtained on a vessel by means of the SAE of myrtle leaf extract.

No free powder precipitate was obtained from runs 1, 2 and 3, but a sticky mixture was obtained, which makes any measurement on it difficult. Based on the study by Reverchon and De Marco, larger particle sizes and non-uniform precipitates are obtained when the operating values do not reach into the supercritical single-phase region of the vapor-liquid equilibrium diagram of the CO_2_-organic solution mixture [47]. This is in agreement with the results obtained in runs performed at low pressure (9 MPa—run 4), where clusters based on solid particles larger than one-micron size stuck together were obtained. Figure 3 shows the powder precipitated on a vessel corresponding to run 5. In general, submicron quasi-spherical particles can be observed when operating at not extremely high pressures and mild temperatures, as in run 5. This probably happens because when operating just above the supercritical conditions of the mixture, its surface tension becomes zero, especially at the end of the mixing process [47].

In our case, when pressure was increased, smaller particle sizes were obtained from the myrtle leaf extracts by SAE. This could be explained by the increasing density and higher solubility, together with other factors, caused by the higher pressure. Furthermore, at a higher pressure, the micro-droplets that originated in the spraying nozzle break into smaller particles [48]. On the other hand, the smallest particle size was obtained when the average pressure level of the design was used (14.5 MPa). It is well known that when conditions are close to the critical point, a minor change in pressure causes considerable changes in the density and solubility of the compounds that are fully related to this density value. In certain situations, always above the critical point, lower pressure and the subsequent lower solubility may lead to a higher supersaturation of the system, which results in the generation of smaller particles. This outcome was observed in the replicate runs 5, 6 and 7 and is in agreement with the results obtained by other authors when investigating the supercritical antisolvent process [49,50,51].

However, temperature increases, on the one hand, lead to a decrease in the solubility of the polyphenols in the solution and, on the other hand, reduced the solvent capacity of the CO_2_. In that case the first effect prevails and thus enhances the maximum supersaturation, so that smaller particles are generated [48]. This could, therefore, explain the smaller particle sizes obtained in tests 10 and 11 when compared against those obtained in tests 8 and 9. 

**Figure 4:**
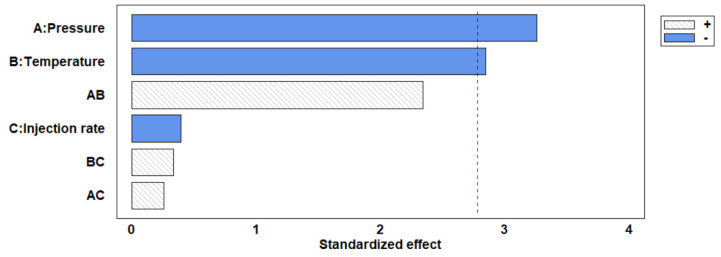
Pareto diagram representing the effect of different parameters on the particle size. “+” (positive effect) “−” (negative effect).

A statistical analysis of the influence on the particles’ size as a result of varying the process variables’ values was performed. The results can be seen in the Pareto chart in Figure 4. It was revealed that the process was dependent on temperature and, above all, pressure, both parameters presenting a significant effect (*p* < 0.05). In this regard, the extract injection rate once the desired pressure and temperature conditions had been reached did not seem to have any obvious effects. This was probably due to the micro-droplets produced by the spray and the mass transfer that took place during the precipitation stage, both of which were suitable to obtain particles of the desired size in most of the experiments. Therefore, it was concluded that the process was mainly controlled by the supersaturation of the compounds present in the extract, as discussed above.

In addition, and in order to prove the repeatability of the process, three replications of the center point of the design were carried out (runs 5, 6 and 7). According to the results, the authors have concluded that the repeatability of the precipitation process was satisfactory, since the differences in phenolic content were below 7%, while the particle sizes obtained never differed by more than 20%.

#### 3.1.2. Total Phenolic Content

The total content of phenolic compounds in the precipitates has been presented in the summary of Table 4. The operational factors that may affect the stability of the phenolic compounds are numerous, such as the presence of oxygen, light exposition, pH, processing times and, especially, temperature, since their degradation increases when this factor rises significantly [52]. In this respect, the lower amount of total polyphenols obtained from runs 4, 10 and 11 could be explained by the oxidation that takes place during supercritical precipitation at high temperatures (328 K). Furthermore, high temperatures may enhance the solubility of these compounds into the ethanol/CO_2_ mixture, which results in their being swept out of the vessel together with the CO_2_ flow [53]. 

**Figure 5:**
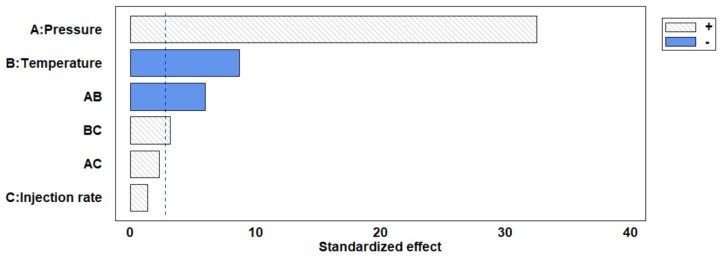
Pareto diagram representing the effect of different parameters on the TPC. “+” (positive effect) “−” (negative effect).

Furthermore, the effect of pressure on the final phenolic content is positively correlated, with the greater yields obtained probably due to the higher solubility and to the enhancement of the mass transfer. This could be confirmed by comparing the results from runs 4 and 11, which were performed at the same temperature. Promising values regarding the phenolic compounds content were obtained when the process was carried out at milder temperature and pressure levels (run 5). In general, based on the results registered for the experiments, higher pressure values and mild temperatures are recommended to achieve the improved precipitations of bioactive compounds by SAE. Figure 5 shows the Pareto chart of the studied variables for the amount of phenolic compounds present in the samples. It can be confirmed that this value is strongly controlled by the pressure of the process, as mentioned above, and to a lesser extent by the temperature in an inverse relationship.

In fact, with regard to the phenolic content of the precipitates, SAE should be considered an effective method, given that the actual TPC content in the precipitate exceeded, in most cases, that found in the starting myrtle extracts (495.43 mg GAE/g dry weight). The obtained results show that the SAE process selectively enriched the precipitates of polyphenol compounds in certain conditions. This fact is probably due to the removal of certain unwanted compounds through the condensing phase in SAE, which results in a higher concentration of phenolic compounds in the precipitated fraction [54,55]. 

#### 3.1.3. Quantification of Polyphenols by Mass Spectrometry

The negative ESI/MS ionization conditions applied for the screening of different compounds allowed the identification of a total of 8 compounds in the myrtle leaf extract and precipitates. The compounds were identified by comparing their retention times and spectra provided by QTof MS with the standards previously studied. Table 5 shows the retention times, molecular formula, and molecular ions [M−H]^−^ of each identified compound. The presence of compounds such as synaptic acid, ferulic acid, myricetin and coumaric acid was also studied, but quantification was not possible.

**Figure 6:**
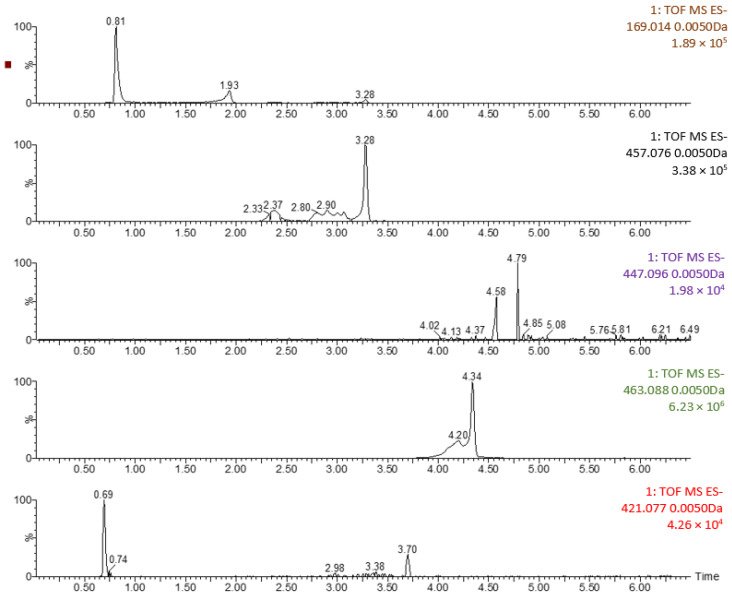
Mass spectra for the identification of compounds in run 8.

The quantification of these compounds was studied on the basis of previous studies that identified them in extracts of myrtle leaves obtained by different processes (maceration, sonication, liquid phase extraction or supercritical fluid extraction, among others) [56,57,58,59]. Figure 6 shows the spectra of run 8 where the peaks corresponding to the different compounds identified can be seen. The rest of the spectra of the extract and the rest of the samples can be seen in the supplementary material (Figures S1–S6). Table 6 shows the individual profiles for the various identified polyphenols; moreover, the concentrations of many of them vary considerably with the experimental conditions of the SAE technique. 

In general, and in decreasing order of concentration, kaempferol -3-glucoside was followed by quercetin, epigallocatatechin gallate, mangiferin, gallic acid, myricetin and vitexin, all of which are widely reported in the literature to be in myrtle leaf extracts. It is worth noting the absence of myricetin, which is abundant in the different extracts described. This may be due to the extraction method or that it was removed together with the CO_2_ phase in the precipitation process by affinity. 

Interestingly, the data are in line with those discussed in the previous section on total phenolic content. The major difference with the total phenolic content is in Run 5, which must be concentrated in different phenolic compounds not identified in this study. When the precipitation process takes place at low to medium temperatures, this minimizes the thermal degradation of polyphenols, resulting in a higher value of these in the final product. On the other hand, increases in process pressure benefit the increase in concentration of these polyphenols, probably due to, among other causes, the increased solvent and compounds with a non-polar character removal in precipitates. 

#### 3.1.4. Antioxidant Activity of Precipitated Particles

**Figure 7:**
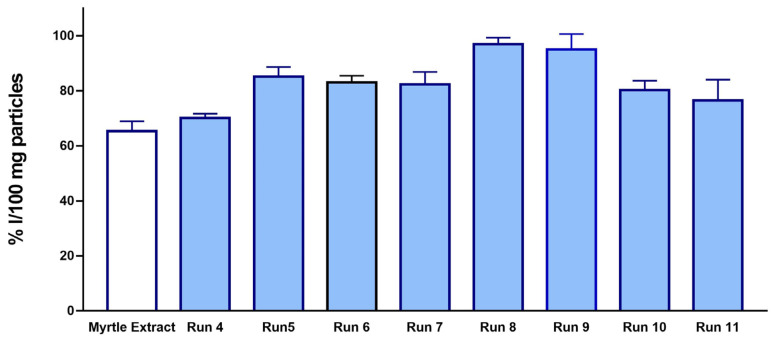
Antioxidant activity of processed particles.

The data on the antioxidant capacity of the precipitates is shown in Figure 7, expressed as the percentage of inhibition of the oxidation per 100 mg of particles. The antioxidant activity of the SAE precipitates of myrtle leaf extracts according to the DPPH oxidation tests were all higher than those of the previously used extracts (65.4% I/100 mg dry weight of extract). This fact supports the high efficiency of the SAE process to obtain potent antioxidant products that not only preserve their original bioactivity capacity, but it is also improved in most cases. This enhanced bioactivity must be closely related to the ability of scCO_2_ to produce precipitated fractions mostly composed of polyphenolic compounds with a marked oxidant capacity, while others are removed. Similar enhancements of the bioactive capacity have been observed when SAE and ethanolic extracts have been applied to other raw materials such as grape seeds [60], mango leaves [32,37], or grape residue [18].

### 3.2. The Antioxidant Properties of Myrtle Particles Attenuate tBHP-Induced Cell Death and Oxidant Stress Injury in RAW 264.7 Cells

**Figure 8:**
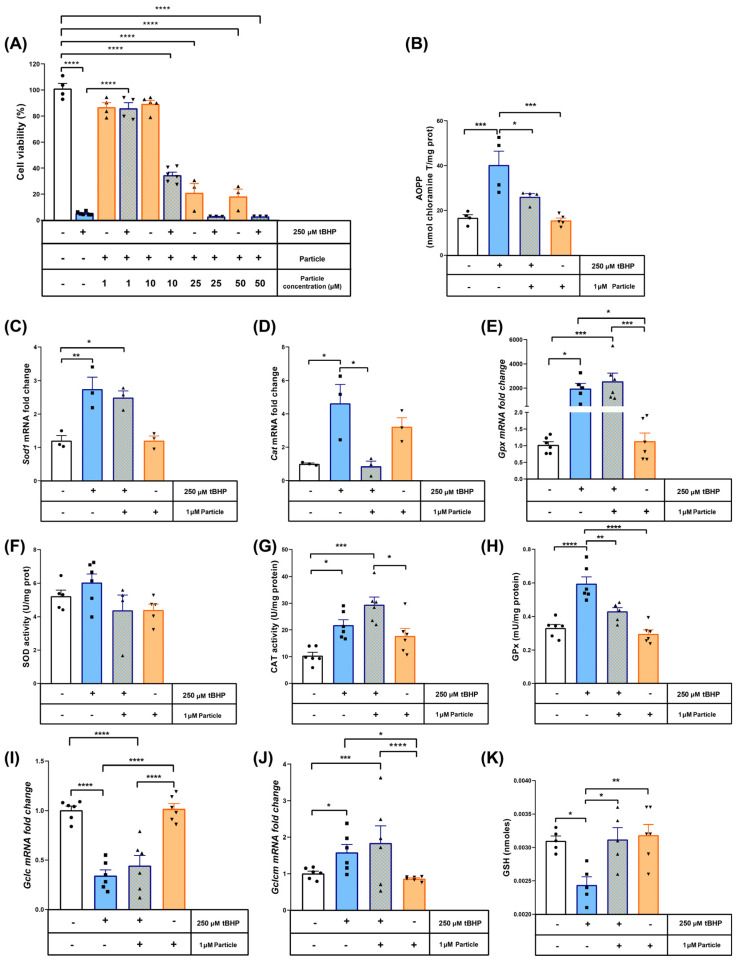
Cytoprotective and antioxidant role of myrtle particles in murine macrophage cell lines. (**A**) Viability of the cells treated with 250 µM tBHP for 1 h according to MTT assay. Cytoprotective effect of the different concentrations of myrtle particles ranging from 1 to 50 µM. Antioxidant properties at a concentration of 1 µM myrtle particles (**B**) Advanced Oxidation Protein Products (AOPPs). qPCR assays for genes encoding free radical scavenging enzymes: SOD1 (**C**), CAT (**D**) and GPX (**E**). The expression values represent four biological replicates and are shown relative to the β-Actin expression as a housekeeping gene. The mRNA levels of the control group for each gene were set at 1. Activity of the antioxidant enzymes superoxide dismutase (**F**), catalase (**G**) and glutathione peroxidase (**H**). (**I**) Reduced glutathione content. mRNA levels of the catalytic (**J**) and modifier (**K**) subunits of the Glutamate-cysteine ligase. The expression values represent four biological replicates and are shown relative to the β-Actin expression as a housekeeping gene. The mRNA levels of the control group for each gene were set at 1. The results are represented as the mean ± SEM. * *p* < 0.05; ** *p* < 0.01; *** *p* < 0.001; **** *p* < 0.0001. “+” (compound added) “–” (not added). Circles: control; squares: 250 µM tBHP added; up triangle: co-treatment with tBHP + particle; down triangle: only particle treatment.

In order to determine the potential cytoprotective effect of the myrtle submicron particles, concentrations ranging from 1 to 50 μM were tested in an MMT assay using murine macrophage cell lines treated with the pro-oxidant agent tBHP. The sample corresponding to the midpoint (Run 5–14.5 MPa, 318 K and 5.5 mL/min), because of its lower particle size and high antioxidant activity (0.46 μm and 85.05%I, respectively), was selected for all of the in vitro tests. As shown in Figure 8A, 250 μM tBHP after 1-h incubation reduced cell viability down to 10%. This cell death rate was fully inhibited when the cells were co-treated for 16 h with 1 μM myrtle particles. Therefore, this concentration was selected for further investigation. Actually, higher particle concentrations did not show any greater cytoprotective effect. Thus, when the concentration was increased to 10 μM, less than 40% of the cells were recovered in comparison to the control test. Higher concentrations (25 and 50 μM) proved to be cytotoxic per se, so that they did not exert any kind of protection against tBHP. 

The antioxidant properties of the myrtle submicron particles were also determined. The incubation of the tBHP caused oxidative damage to the proteins, as inferred by the 2-fold increase in AOPPs, which was prevented by 1 μM myrtle particles (Figure 8B). In order to gain a better insight of the protective mechanism, the mRNA levels of the main antioxidant enzymes were measured. Thus, we found a significant increase in the expressions of Sod1, Cat or Gpx induced by the tBHP (Figure 8C–E). The treatment with myrtle particles only reduced the expression of Cat back to control levels, with no apparent effects on the Sod1 or Gpx expressions. In order to evaluate if these changes in mRNA levels had an impact on the proteins’ function, the enzymatic activity of CAT, SOD and GPx in protein extracts were determined (Figure 8F–H).

As expected, no differences were observed in the SOD activity with any of the treatments, since tBHP is not a superoxide generator. However, both CAT and GPX activities increased with the pro-oxidant treatment. The myrtle precipitated particles reduced the activity of GPx down to the levels of the control test. This fact allowed us to quantify the amount of reduced glutathione, as it is a GPx cofactor and the main water-soluble antioxidant (Figure 8I). The tBHP treatment significantly depleted the GSH content, but the coincubation with myrtle particles resulted in an antioxidant-preserving effect that kept GSH levels down to the control test levels. The expression of the catalytic subunit of the glutamate-cysteine ligase, which is responsible for the GSH synthesis, was downregulated in both tBHP-treated groups, regardless of the addition of myrtle, while the modulator subunit, on the other hand, was also upregulated in both groups (Figure 8J,K). This suggests that the antioxidant properties exhibited by the myrtle precipitated particles obtained by SAE are explained by the prevention of GSH consumption, given that its synthesis remains unaltered.

Taking into account the promising results obtained in terms of bioactive, antioxidant and particle size properties, the products obtained could be a viable alternative for treatments where oxidative stress is present, such as obesity, diabetes, hypertension, or atherosclerosis with vascular risk factors [61]. In addition to these results, the RAW 264.7 cell line is related to research on cancer and cardiovascular problems, so the obtained results in this area could be investigated further [62,63].

## 4. Conclusions

The effectiveness of Supercritical CO_2_ Antisolvent Extraction techniques to obtain highly antioxidant submicron particles from *Myrtus communis* L. extracts has been demonstrated. Thus, submicron particles with sizes ranging from 0.42 to 1.32 μm were obtained. The particle sizes presented a decreasing trend as pressure and temperature were increased. No conclusions were reached with regard to the influence of the extract injection rate. The process proved its suitability to produce phenolic compound concentrates with increased antioxidant activity that could even reach oxidation inhibition rates up to 97%. With regard to particle size and precipitates’ bioactivity, the best SAE conditions, within the studied range, for the extraction of myrtle leaves using ethanol as a solvent have been established at 14.5 MPa and 318 K (Run 5). Different polyphenols have been identified both in the extract and in the precipitates. A number of in vitro experiments on macrophage cell lines treated with tBHP corroborated the high cytoprotective capacity of the particles that conformed the precipitates obtained by subjecting *Myrtus communis* L. extracts to the SAE method investigated in this study, even when used at a low concentration (1 μM).

## Figures and Tables

**Table 1:** Summary of the SAE experiments completed.

Runs	P (MPa)	T (K)	Injection (mL/min)
1	9	308	3
2	9	308	8
3	9	328	3
4	9	328	8
5	14.5	318	5.5
6	14.5	318	5.5
7	14.5	318	5.5
8	20	308	3
9	20	308	8
10	20	328	3
11	20	328	8

**Table 2:** The analytical characteristic for determination of myrtle leaf compounds.

Compounds	Linear Equation	R^2^
Mangiferin	y = 47.0275 × x	0.9999
Quercetin	y = 50.9071 × x	0.9984
Myricetin	y = 25.8573 × x	0.9991
Kaempferol -3-glucoside	y = 114.385 × x	0.9833
Kaempferol	y = 59.5196 × x	0.9998
Sinapic acid	y = 63.1047 × x	0.9955
Vitexin	y = 97.6308 × x	0.9999
Ferulic acid	y = 30.0698 × x	0.9820
Epigallocatatechin gallate	y = 39.242 × x	0.9897
Coumaric acid	y = 74.3687 × x	0.9877
Gallic acid	y = 11.284 × x	0.9943

**Table 3:** Quantitative real-time PCR oligonucleotides.

	Forward	Reverse
β-Actin	5′-AGGTGACAGCATTGCTTCTG-3′	5′-GCTGCCTCAACACCTCAAC-3′
Cat	5′-GTGCATGCATGACAACCAG-3′	5’-TGAAGCGTTTCACATCTACAGC-3′
Gclc	5′-TTGTCGCTGGGGAGTGATTT-3′	5′-TGATCCTAAAGCGATTGTTCTTCAG-3′
Gclcm	5′-TGACTCACAATGACCCGAAAGA-3′	5′-CCCCTGCTCTTCACGATGAC-3′
GPx1	AGGCGGGACCCTGAGACTTA-3′	5′-ATCACGTGGCATCGCTTTCT-3′
Sod1	5′-CAGGACCTCATTTTAATCCTCAC-3′	5′-CCCAGGTCTCCAACATGC-3′

**Table 4:** Particle size and TPC obtained through SAE.

Runs	P (MPa)	T (K)	Injection (mL/min)	Particle Size (μm)	TPC (mg GAE/g Precipitate)
1	9	308	3	-	-
2	9	308	8	-	-
3	9	328	3	-	-
4	9	328	8	1.32 ± 0.41	347.62 ± 5.67
5	14.5	318	5.5	0.46 ± 0.25	534.57 ± 18.53
6	14.5	318	5.5	0.52 ± 0.43	530.42 ± 2.45
7	14.5	318	5.5	0.42 ± 0.17	525.37 ± 10.23
8	20	308	3	0.95 ± 0.36	556.34 ± 23.47
9	20	308	8	0.74 ± 0.25	552.47 ± 14.67
10	20	328	3	0.67 ± 0.23	452.12 ± 1.45
11	20	328	8	0.62 ± 0.63	370.58 ± 3.56

TPC: total phenolic content.

**Table 5:** Quantified compounds by UPLC-MS in myrtle leaf precipitates.

Compound	T_R_ (min)	Formula	[M–H]^−^
Mangiferin	3.7	C_19_H_18_O_11_	421.077
Quercetin	4.35	C_21_H_20_O_11_	463.088
Kaempferol-3glucoside	4.76	C_21_H_18_O_12_	447.096
Kaempferol	5.61	C_15_H_10_O_6_	285.040
Vitexin	4.31	C_21_H_20_O_10_	431.098
Epigallocatatechin gallate	3.28	C_22_H_18_O_3_	457.076
Gallic acid	1.92	C_7_H_6_O_5_	169.014

**Table 6:** Chemical composition of myrtle leaf extract and precipitates.

Compound	Extract	Run 4	Run 5	Run 8	Run 9	Run 10	Run 11
Mangiferin	34.6 ± 2.4	4.5 ± 3.7	83.9 ± 23.6	172.49 ± 22.1	3.9 ± 0.3	16.7 ± 1.4	2.5 ± 0.3
Quercetin	98.9 ± 2.6	NQ	36.2 ± 6.1	94.97 ± 27.9	43.5 ± 4.6	1.4 ± 0.1	28.4 ± 3.7
Kaempferol 3-glucoside	168.4 ± 14.6	16.0 ± 4.8	NQ	157.31 ± 11.8	107.4 ± 23.6	NQ	NQ
Kaempferol	4.9 ± 2.3	NQ	NQ	7.34 ± 6.9	31.6 ± 6.7	NQ	NQ
Vitexin	0.3 ± 0.2	0.3 ± 0.1	0.3 ± 0.1	0.4 ± 0.2	NQ	0.3 ± 0.1	NQ
Epigallocatatechin gallate	38.2 ± 4.5	0.8 ± 1.7	1.5 ± 0.4	43.9 ± 9.5	24.6 ± 2.1	1.0 ± 0.4	0.5 ± 0.1
Gallic acid	22.8 ± 6.8	2.5 ± 0.4	6.1 ± 2.1	28.1 ± 2.4	31.7 ± 6.9	15.6 ± 2.0	0.8 ± 0.1

Results expressed in mg/g of precipitate or dry weight in the case of the extract; NQ: No quantified.

## Data Availability

The data presented in the study are available on request from the corresponding author.

## Supplementary Materials

The following supporting information can be downloaded at: https://www.mdpi.com/article/10.3390/antiox12020530/s1, Figure S1: Mass Spectra for myrtle leaf extract; Figure S2: Mass Spectra for Run 4; Figure S3: Mass Spectra for Run 5; Figure S4: Mass Spectra for Run 9; Figure S5: Mass Spectra for Run 10; Figure S6: Mass Spectra for Run 11.
