# Supplementary material for: Generation of Highly Antioxidant Submicron Particles from Myrtus communis Leaf Extract by Supercritical Antisolvent Extraction Process
Source: Antioxidants (Basel). 2023 Feb 20;12(2):530. doi: 10.3390/antiox12020530 (PMC9951993; doi:10.3390/antiox12020530)
Supplement: Supplementary file 1 [file antioxidants-12-00530-s001.zip › antioxidants-2194649-supplementary.pdf]

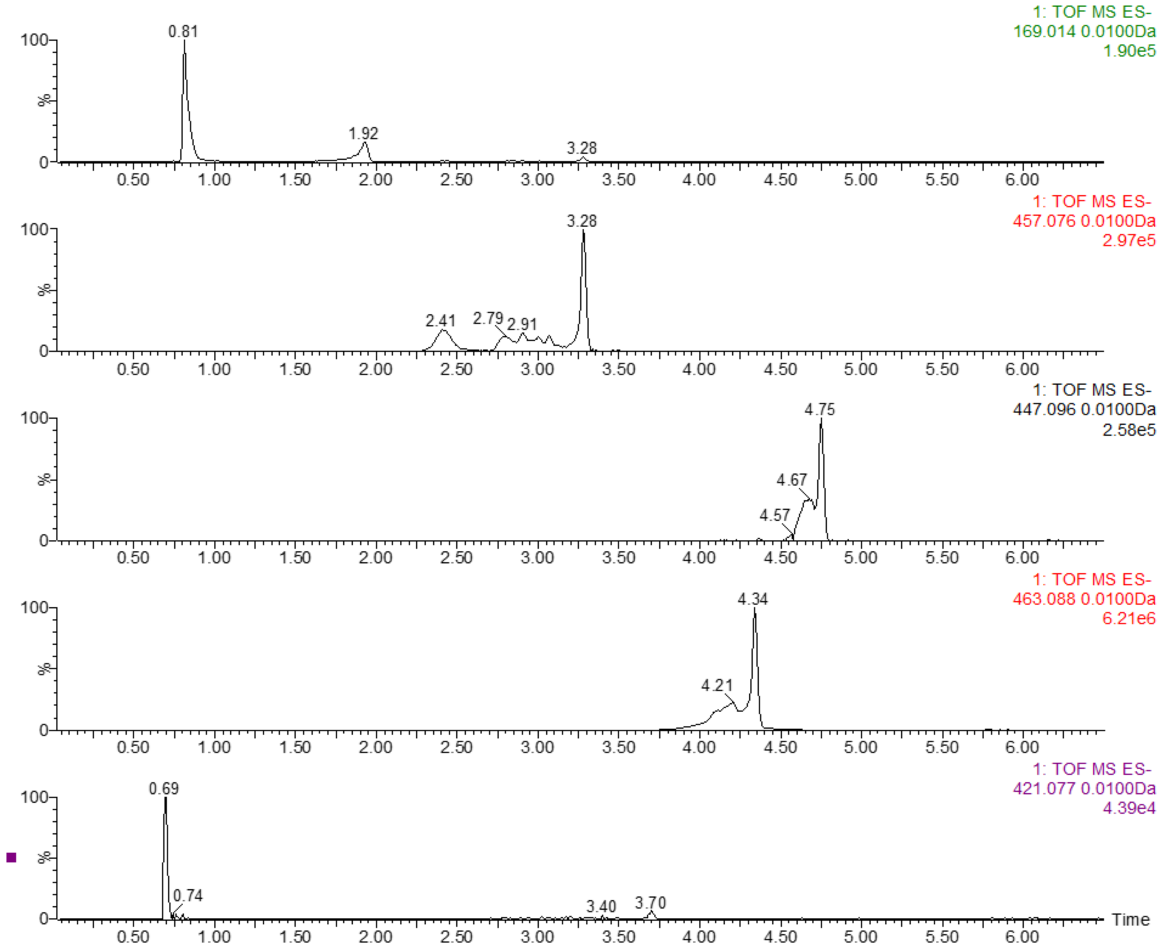

Supplementary Figure S1. Mass Spectra for myrtle leaf Extract

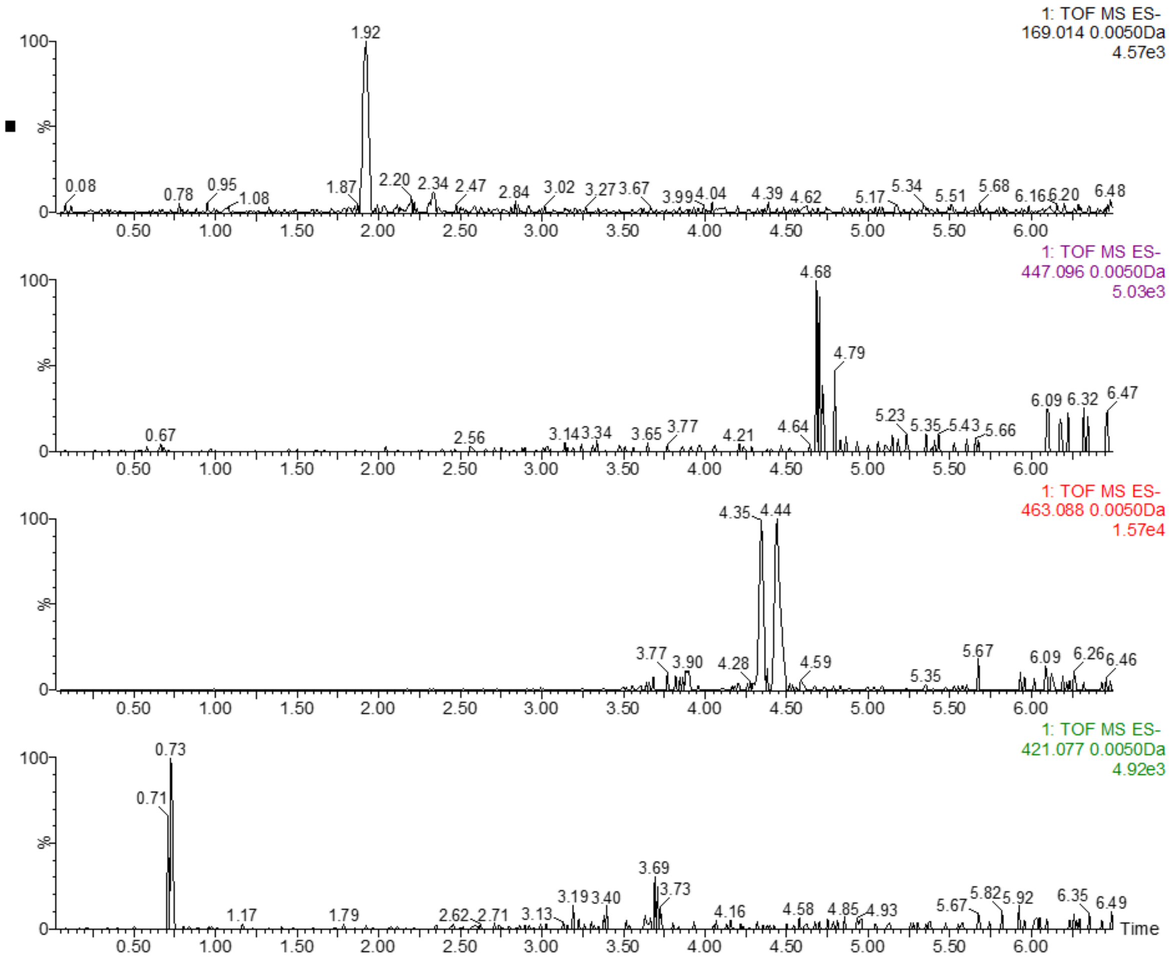

Supplementary Figure S2. Mass Spectra for Run 4.

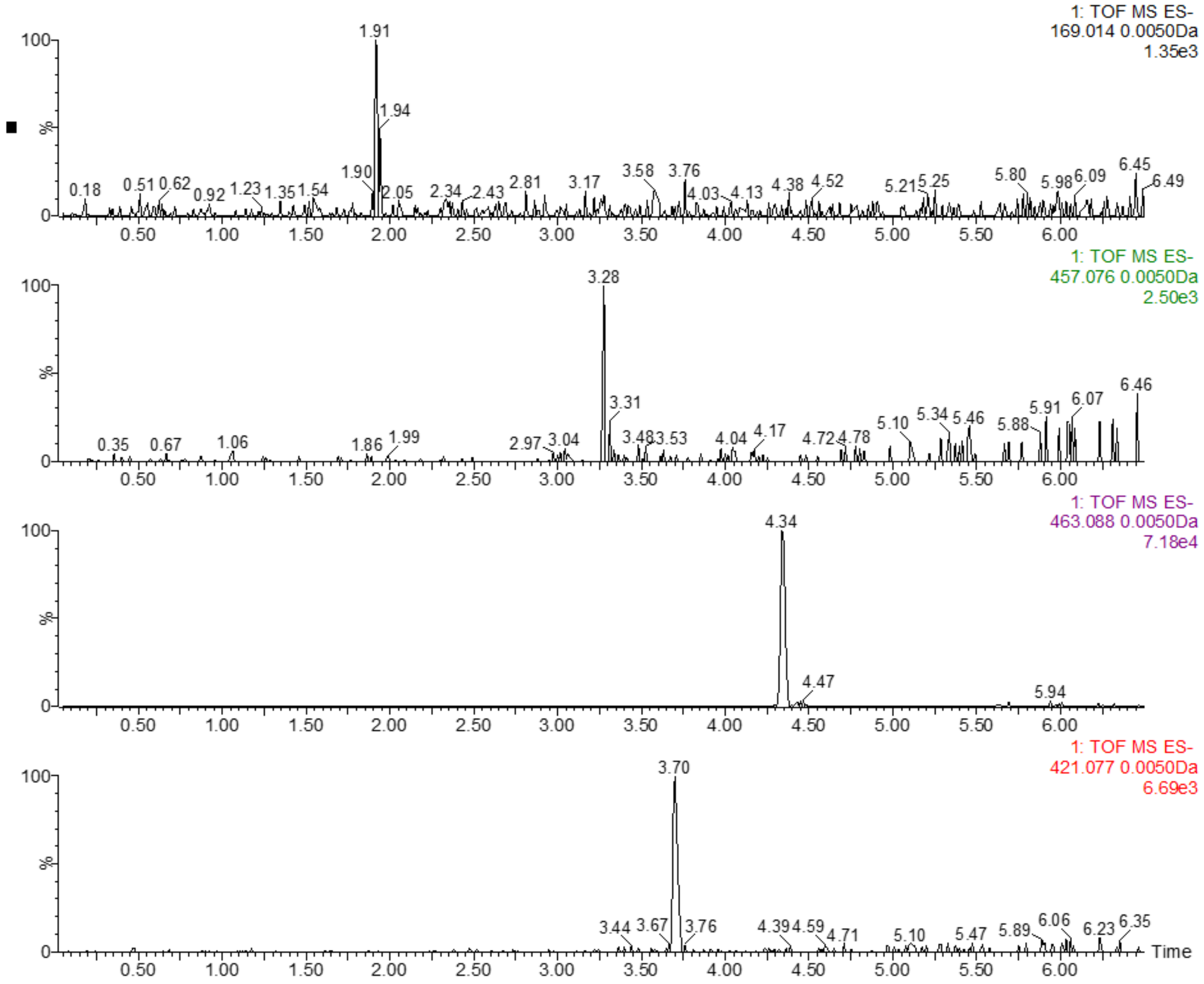

Supplementary Figure S3. Mass Spectra for Run 5.

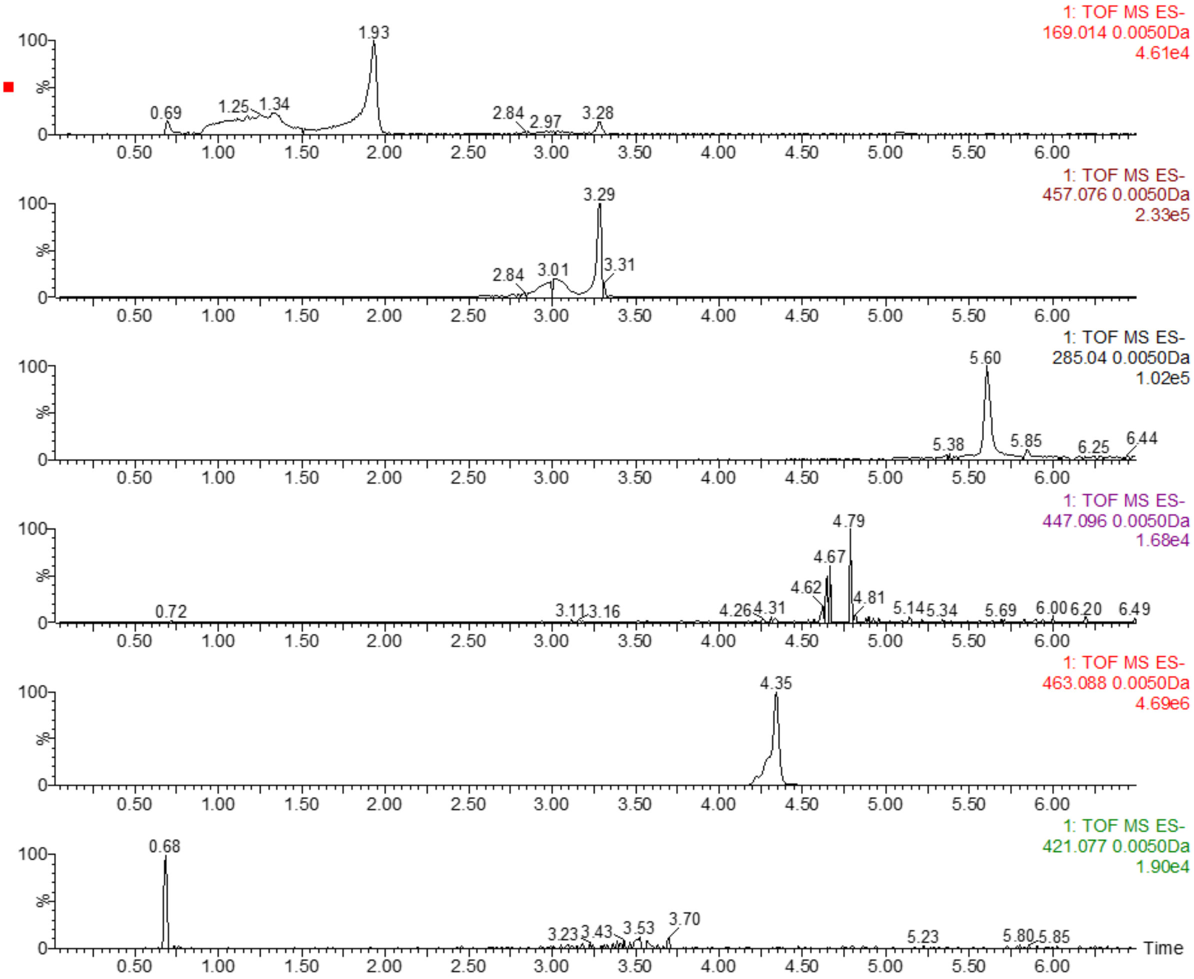

Supplementary Figure S4. Mass Spectra for Run 9.

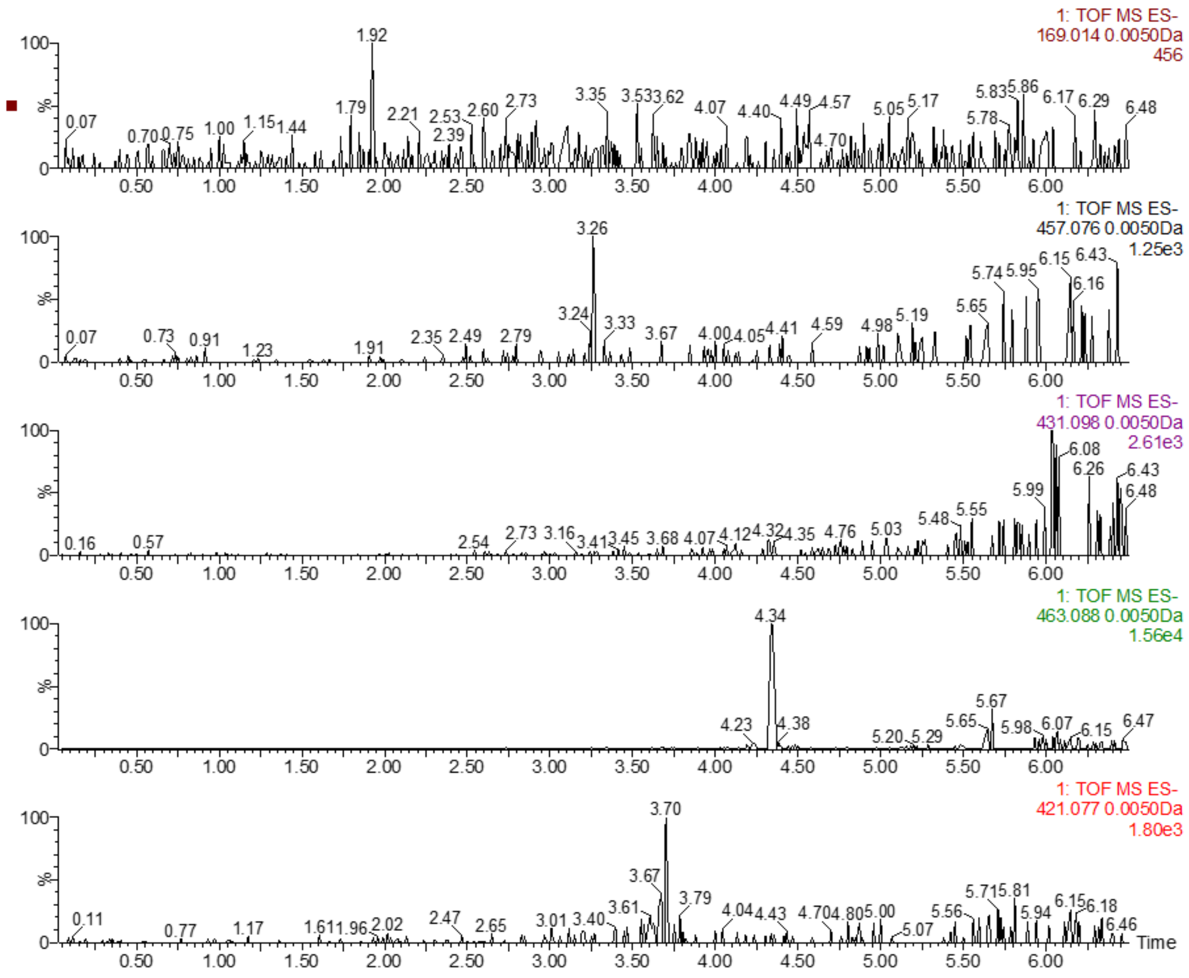

Supplementary Figure S5. Mass Spectra for Run 10.

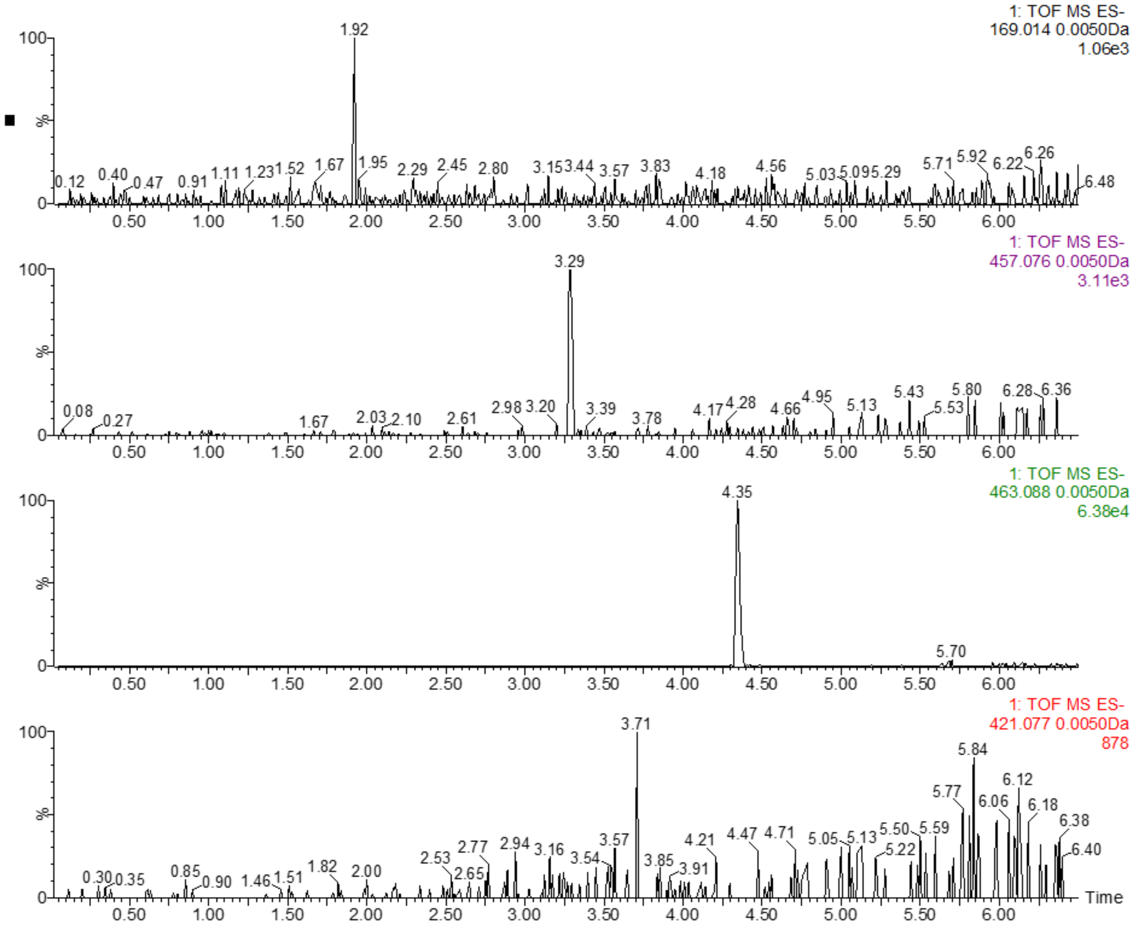

Supplementary Figure S6. Mass Spectra for Run 11.
